# Rethinking village malaria workers in Cambodia: Perspectives from the communities, programme managers, and international stakeholders

**DOI:** 10.1371/journal.pgph.0003962

**Published:** 2024-12-11

**Authors:** Long Heng Orng, Monnaphat Jongdeepaisal, Panarasri Khonputsa, Lek Dysoley, Siv Sovannaroth, Thomas J. Peto, James J. Callery, Christopher Pell, Richard J. Maude, Marco Liverani

**Affiliations:** 1 Mahidol Oxford Tropical Medicine Research Unit, Faculty of Tropical Medicine, Mahidol University, Bangkok, Thailand; 2 Centre for Tropical Medicine and Global Health, Nuffield Department of Medicine, University of Oxford, Oxford, United Kingdom; 3 National Center for Parasitology, Entomology and Malaria Control, Phnom Penh, Cambodia; 4 Amsterdam Institute for Global Health and Development (AIGHD), Amsterdam, The Netherlands; 5 Department of Global Health, Amsterdam University Medical Centers—Location Academic Medical Center, Amsterdam, The Netherlands; 6 Centre for Social Science and Global Health, University of Amsterdam, Amsterdam, The Netherlands; 7 The Open University, Milton Keynes, United Kingdom; 8 Department of Global Health & Development, London School of Hygiene & Tropical Medicine, London, United Kingdom; 9 School of Tropical Medicine and Global Health, Nagasaki University, Nagasaki, Japan; 10 Faculty of Public Health, Mahidol University, Bangkok, Thailand; Menzies School of Health Research, AUSTRALIA

## Abstract

Since the early 2000s, malaria cases in Cambodia have declined steadily. Village malaria workers (VMWs) have played a critical role in reducing malaria transmission and progress towards malaria elimination. To prevent malaria re-establishment, however, implementation strategies need to consider carefully the changing healthcare needs in the communities as well as challenges to, and opportunities for, programme adaptation. We conducted in-depth interviews with a diverse range of stakeholders involved in the planning or implementation of the VMW programme in Cambodia, to elicit their views and experiences about health priorities in the communities, the public health value of VMWs and community-based care, and prospects for future programme development. Respondents included managers and implementers involved in the VMW programme at the central and provincial level (n = 9), technical officers at international agencies in Cambodia (n = 7), international stakeholders in non-governmental and research organisations based in Cambodia or other countries in the region (n = 5), as well as VMWs (n = 10), and community members (n = 16) in six endemic communes of Kravanh District, Pursat Province. In Kravanh, we also conducted four focus group discussions with 19 community members who had previous experience of malaria. The qualitative dataset was analysed using a thematic approach. VMWs, particularly mobile malaria workers tasked with active case detection among forest workers, were deemed necessary to maintain effective malaria control. However, there was a clear demand in the communities for additional services including treatment for common illnesses, monitoring of blood pressure and blood sugar levels, and relief of general symptoms through medication, such as for fever, headache, and stomach pain. Programme managers and international stakeholders agreed that the VMW programme needs a rethinking of the current implementation model to ensure continued uptake, relevance, and motivation of VMWs. Suggestions for add-on activities included adoption of new tests for febrile illnesses such as dengue and chikungunya, and screening for the prevention and monitoring of non-communicable diseases. There was emphasis on the needs for more sustainable financing mechanisms and integration with the existing community health infrastructure. The potential expansion of VMW services will benefit from the continued involvement of external donors and partners for technical and financial support. However, the implementation strategy should consider since the outset opportunities for enhanced local ownership and health system integration. To maintain domestic political momentum and access new potential sources of domestic funding, further programme development should align with national health priorities and the ongoing process of administrative decentralisation, while being responsive to changing public health needs within the communities.

## Introduction

Countries in Southeast Asia continue to make progress in malaria control and are close to eliminating *Plasmodium falciparum* infection [1]. In Cambodia, malaria incidence has decreased dramatically, from 106,905 cases in 2011 to 4,329 confirmed cases in 2021, with no malaria-related deaths since 2018 [2]. Between January and March 2024, the Cambodian National Center for Entomology, Parasitology and Malaria Control (CNM) reported only 176 cases—a 64% decrease compared to the same period in 2023—and 92% of them with *Plasmodium vivax*. Faced with stretched healthcare budgets and competing priorities when approaching elimination, it might be tempting to de-prioritize malaria as a public health threat and allocate fewer resources to malaria control. However, achieving sustainable elimination requires a concerted and continuous effort, particularly among forest workers and mobile population groups due to their increased exposure to malaria risk, transient living conditions, and limited access to healthcare services [3, 4]. Community health workers have provided these population groups with essential services for malaria prevention, testing, and treatment. In Cambodia, the largest community-based intervention for malaria control is the Village Malaria Workers (VMW) programme, established in 2004 by the Ministry of Health with support from the Global Fund and other international organisations [3, 4]. Initially focused on case management in endemic villages, this programme was later expanded to include distribution of long-lasting insecticidal nets (LLINs) and targeted activities to reach out to mobile populations and forest workers. In 2016, the programme was nearly suspended due to changes in the disbursement policy that affected payments of VMWs, with a substantial reduction in testing and treatment followed by a resurgence in malaria cases [5]. Consequently, support for VMWs was reinstated and their involvement was identified as a key element in the national strategy to eliminate *P*. *falciparum* by 2025 and the wider regional strategy for malaria elimination in the Greater Mekong Subregion by 2030 [2, 6].

As malaria declines, identifying and treating the remaining cases is crucial to achieving and maintaining elimination, in Cambodia and the wider region [7–9]. In many locations, however, people are aware that malaria has reduced to almost zero and is generally not the cause of fever and related symptoms. This perception leads many to skip consultation with VMWs, who are trained to diagnose and treat only malaria. As a result, malaria cases may go undiagnosed or are diagnosed too late, which can lead to more severe illness, prolonged transmission, and setbacks in malaria control efforts [10].

One strategy to address this issue, and sustain investment in malaria elimination, is to assign new primary care tasks to VMWs, in addition to their established malaria-related services. However, to ensure that programme implementation is effective and sustainable, the offer of services provided must consider care needs and preferences in the communities as well as the wider health system and policy contexts. Drawing on qualitative data from in-depth interviews and focus group discussions, this article presents the views and experiences of stakeholders in Cambodia regarding current health issues in remote and rural communities and prospects for the development of community-based services to address them. A qualitative study of community needs and care options in Cambodia is valuable as it provides in-depth insights into the lived experiences, social dynamics, and health sector issues that shape healthcare practices. By capturing the voices and perspectives of domestic and international stakeholders, the study can inform more appropriate and effective healthcare policies and interventions.

## Methods

### Ethics declaration

The study was approved by the National Ethics Committee for Health Research reference: 318, 24^th^ December 2021, and Oxford Tropical Research Ethics Committee (OxTREC) approval reference: 535–21, 24^th^ August 2021. All respondents gave written informed consent to participate in the interviews and to be audio-recorded. Respondents were informed that they had no obligations to participate and that there would be no consequences for them should they decide not to participate. They were also informed that their decision to participate would be kept confidential. Participants were compensated for their time (5 USD) and travel expenses (up to 10 USD).

### Study design

This study was part of two multi-country research programmes across Southeast Asia that aimed to better understand health concerns and health needs in remote and rural communities and to conduct various assessments, reviews and implementation trials of scalable, high-impact interventions. These included an assessment of VMW programmes across the Asia-Pacific funded through the Regional Artemisinin-resistance Initiative 3 Elimination (RAI3E) by the Global Fund to Fight AIDS, Tuberculosis and Malaria [10–12], and a feasibility study of non-malarial febrile illness management at community level through the South and Southeast Asian Community-based Trials Network (SEACTN) funded by the Wellcome Trust [13]. This article presents findings from a qualitative study, which included in-depth interviews (IDIs) and focus group discussions (FGDs) with stakeholders in the communities receiving and providing the malaria services and those involved in planning or implementing VMW service provision in Cambodia to elicit their views and perspectives about current challenges, opportunities, and prospects for future programme development.

### Study context

**Fig 1:**
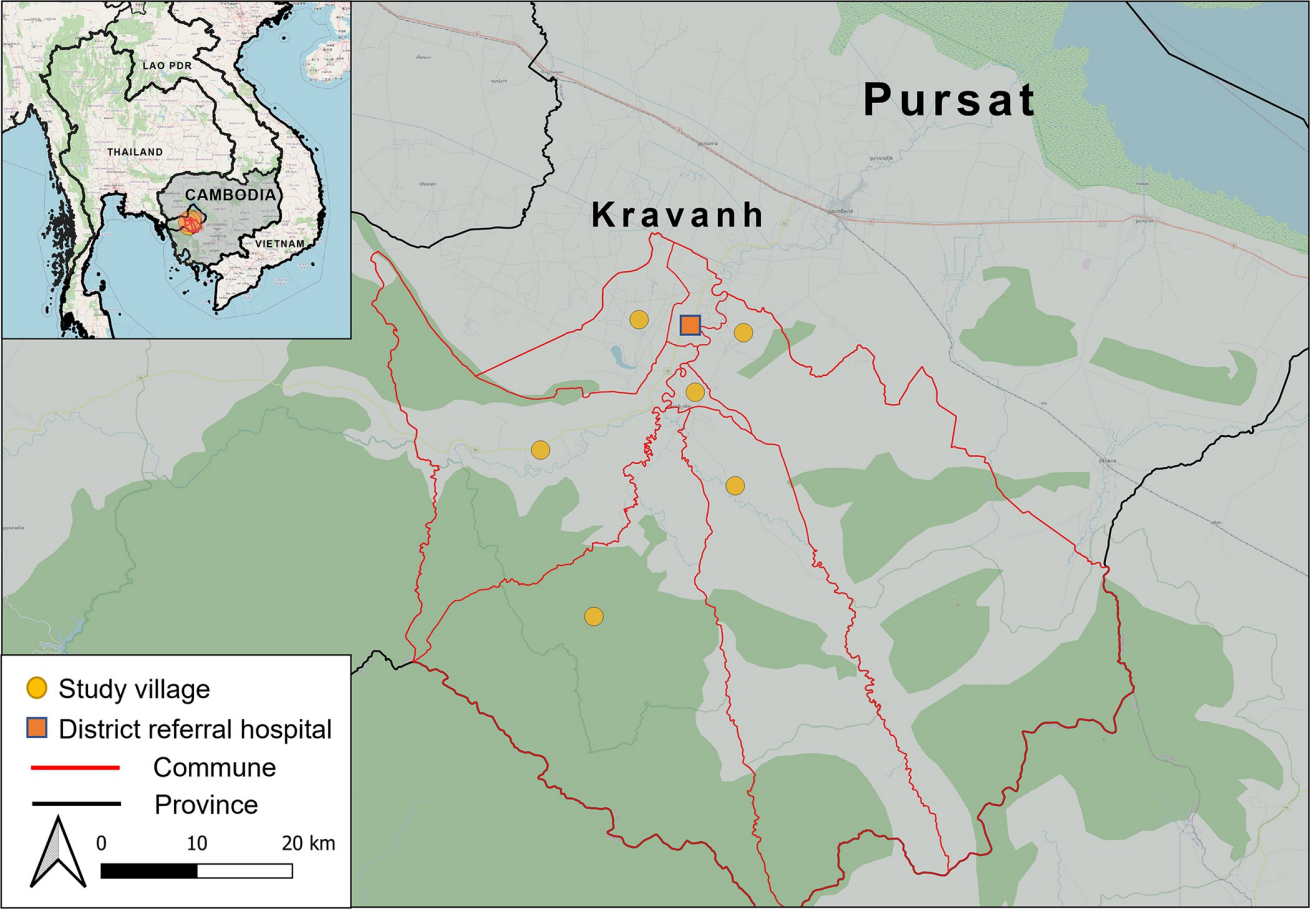
Study provinces and locations of study villages in Cambodia. The map was created using QGIS software version 3.26 (Buenos Aires) and contains a basemap from OpenStreetMap (https://www.openstreetmap.org/#map=6/54.91/-3.43), which is made available under the Open Database License (https://www.openstreetmap.org/copyright). National and provincial administrative boundaries were from Global Administrative Areas version 3.6 (https://gadm.org/download_country.html). Locations of the study sites and district referral hospital were collected on KoboToolbox.

Pursat Province is located in western Cambodia, along the border with Thailand, in an area characterised by the densely forested Cardamom mountains and wildlife sanctuaries (**Fig 1**). Kravanh is 1 of 5 districts in Pursat province and includes 7 communes and 55 villages. According to reports from the Ministry of Health, Kravanh had >40 malaria cases per 1,000 population and an annual blood examination rate of over 20% in 2019. Since then, the number of cases has reduced significantly. From January to March 2024, 176 malaria cases (162 *P*. *vivax* and 2 *P*. *falciparum*) were reported nationally and only one *P*. *vivax* case was identified in Kravanh district [14]. Our study was conducted in 6 malaria endemic communes located at variable distances from health facilities among which, 4 reported confirmed malaria cases in 2019 and 2020 [15].

In addition to primary care centres and the Phnom Kravanh Operational District (OD), a variety of community-based programmes are active in this area, managed by local non-governmental organisations (NGOs) and international organizations (IOs) such as the consulting firm University Research Co (URC) [16, 17] and other organisations implementing the U.S. President’s Malaria Initiative [18]. The VMW programme is managed by the CNM in collaboration with the provincial health departments (PHDs), ODs and health centres. According to the guidelines, VMWs must receive annual training and supervision from ODs and health centre staff on malaria diagnosis and case management, counselling and health education, and case reporting for disease surveillance. Regarding treatment, VMWs are required to follow the protocol on directly observed therapy. *P*. *falciparum* patients should be taking artemisinin-based combination therapy on days 1 and 2, ideally in the presence of VMWs, but when this is not possible, the VMWs should visit the patient on day 3 to confirm that the treatment was completed. For *P*. *vivax* cases, VMWs should conduct a follow-up visit on day 3, 7, and 14 to monitor the patients’ treatment adherence and observe any side effects of primaquine treatment. Mobile malaria workers (MMWs) are tasked with active malaria case detection among mobile and migrant populations, and additional activities such as chemoprevention among forest workers [19]. VMWs/MMWs receive monthly stipends and financial incentives, including incentives to perform specific activities such as outreach and case investigations, although the incentive structure and level vary widely depending on the implementing organisation [20]. All VMWs/MMWs are required to participate in monthly meetings with supervisors to discuss their work, deliver reports and replenish supplies. At the time of the study, there were 71 active VMWs and 16 MMWs in Kravanh District, according to the list in the operational district, covering a population of 50,763 according to the 2019 national census [21]. Nationwide, as indicated by the latest CNM report in 2020 [19], there were 5,968 VMWs active in 2,984 villages (with > 5 malaria cases per 1000 population) and 637 MMWs.

In addition to VMWs, these villages are also served by Village Health Support Groups (VHSG)—a community-based network of volunteers overseen by the Ministry of Health, who work closely with health centres to promote health education and facilitate access to health facilities. The head of the VHSG is elected by the community, and appoints other members, typically with a ratio of 1 volunteer for every 10–50 households. A comparative table of governance, functions, and implementation of VMWs, MMWs and VHSG is provided in Table 1.

**Table 1:** Comparative table of governance, functions, and implementation of community-based health services in Cambodia.

	Village Health Support Groups	Village Malaria Workers	Mobile Malaria Workers
**Governance**	Ministry of Health (National Centre for Health Promotion); Provincial Health Departments (PHDs); local communities	Ministry of Health (National Center for Parasitology, Entomology and Malaria Control (CNM); PHDs; local communities; RAI Regional Steering Committee; development partners	
**Funding**	Government of Cambodia	Global Fund to Fight AIDS, Tuberculosis and Malaria; Bill & Melinda Gates Foundation; other international donors	
**Incentives**	Exemption of user fees at public health facilities; irregular cash transfers	Monthly stipend; per diem when attending meetings at health facilities	Monthly stipend; per diem when attending meetings at health facilities
**Tasks**	Data collection and reporting; health information; maternal and child health promotion; support to health centres in the event of emergencies, such as disease outbreaks or natural disasters, by providing first aid, disseminating information, and coordinating with health authorities	Malaria testing and treatment according to national guidelines; record keeping and reporting; referral of patients with recurring or severe symptoms; management and distribution of LLINs; contribution to health education and the delivery of programmes such as mass screening and treatment campaigns	Active identification and contact tracing of malaria cases in proximity of forests, at least twice a month; malaria testing of all suspected cases and treatment according to national guidelines; distribute “forest packs” with mosquito repellant and LLINs; offer medications to negative malaria cases, such as paracetamol and mebendazole; record keeping and reporting; referral of patients with recurring or severe symptoms; contribution to health education and the delivery of programmes
**Management and implementation**	PHDs; operational districts (ODs); health centres	PHDs; ODs; health centres; national and international NGOs (e.g. Care International; Malaria Consortium; Population Service International; University Research Co.)	
**Technical support**	World Health Organization	World Health Organization; United States Agency for International Development	

Note: Artesunate-Mefloquine (AS-MQ) and single low dose primaquine are the first line therapy for *P*. *falciparum* and *P*. *vivax* patients, while patients with *P*. *vivax* and mixed infections are treated with primaquine (PQ) in accordance with the results of Glucose-6-Phosphate Dehydrogenase (G6PD) deficiency testing, performed to prevent hemolytic anemia in individuals with G6PD deficiency. The provision of PQ and G6PD testing are only available at health centres by the health centre staff in Cambodia.

### Study participants and sampling

For the IDIs, participants were selected to capture the diversity of perspectives of those involved in community-based health care in Cambodia. Following a preliminary stakeholder mapping, based on the review of documents and consultations, summarised in Table 1, we approached and invited to interview representatives of different categories, including: planners and managers of the VMW programme, working in the CNM and other central departments of the Ministry of Health; implementers at the national and sub-national levels, employed in the Ministry of Health or NGOs; and international stakeholders from WHO, research organisations and other agencies involved in programme implementation. In the communities, we aimed to interview VMWs and adult community members, selecting at least one VMW in each of the six communes.

Programme managers and international stakeholders were approached using the professional network of researchers involved in this project, and others were recruited by snowball sampling. In the communities, the first author (LHO) randomly approached residents at their homes, walking through the villages without following a predetermined sampling frame. A group of community members, some of whom participated in the interviews, were later invited to join a focus group discussion.

### Data collection

Interviews were conducted in-person at participants’ homes, in communal spaces, workplaces or online. FGDs were conducted in-person at a public venue in their village or nearby health centre. Semi-structured topic guides for the IDIs and FGDs were developed based on previous studies of community health [22, 23]. They were designed to encourage discussion of priorities in the communities as well as the implementation of community care, including governance, financing and the relation with the wider health system (**S1 Appendix**) [24]. The topic guides were pilot tested with the initial respondents and reviewed for correct translation and clarity. Interviews across all categories of stakeholders and FGDs focused on (1) perceptions about malaria, other health concerns, and access to services in remote and rural communities; and (2) views and experiences about the implementation of the VMW programme. In interviews with programme managers and international stakeholders (3) prospects for the development of community-based care in Cambodia were also examined, considering governance, priorities and financing. The interview guides were iteratively reviewed throughout the data collection period in light of emerging findings.

Most participants were interviewed in Khmer by LHO although some online interviews with planners and implementers were conducted in English and involved MJ and ML. The sample size for the interviews with community members was determined by data saturation. Data collection was completed between March 15th and May 2nd, 2022. The IDIs and FGDs lasted typically between 20–50 minutes and 40–60 minutes respectively, with no participant withdrawals.

### Data processing and analysis

Recorded interviews and FGDs were transcribed, translated into English and imported into NVivo version 12 (QSR International), for qualitative thematic analysis. The analysis aimed to identify themes around the key domains of our investigation defined above, although the coding process was flexible to allow the researcher to identify emerging themes. The consolidated criteria for reporting qualitative research (COREQ) were consulted to ensure comprehensive reporting of qualitative data from interviews and focus groups conducted in this study [25].

## Results

In total, IDIs were conducted with 21 health sector stakeholders, including VMW programme managers and planners at the central and provincial level of the Ministry of Health (n = 9), technical officers at international agencies in Cambodia (n = 7), international stakeholders in non-governmental and research organisations based in Cambodia or other countries in the region (n = 5). In the communities, we interviewed 10 VMWs and 16 community members, located at variable distances from the nearest public health facility (ranging from 20 to 60 minutes by motorbike or car); in addition, we conducted 4 FGDs involving a total of 19 participants, as summarised in **Table 2**. In the sections below, we present the key findings from the IDIs and FGDs, illustrated by quotations and organised under the following headings: (1) perceptions about malaria, other health concerns, and access to services; (2) views and experiences about the implementation of the VMW programme; (3) prospects for the development of community-based care in Cambodia.

**Table 2:** Interviews (n = 26) and focus group discussions (n = 4) in the communities.

	Interviews	Focus group discussions
Village 1	2 VMWs, 7 community members	5 community members
Village 2	2 VMWs	
Village 3	2 VMWs, 5 community members	4 community members
Village 4	2 VMWs, 2 community members	
Village 5	1 VMW, 2 community members	5 community members
Village 6	1 VMW	5 community members

### Malaria and other health concerns

#### Forest workers and malaria

Community members in Kravanh district were farmers who visited the forest regularly or occasionally, spoke Khmer and had Cambodian nationality. Most of those interviewed had malaria in the past but not recently. They were aware of only a few recent cases in their communities. In Kravanh, interviewed VMWs reported that they recently tested people for malaria, typically 2–5 persons per month using malaria rapid diagnostic test (RDT) and that none of them was positive. One VMW mentioned she had not referred a malaria patient to the hospital for more than 2 years.

Respondents explained that the declines in malaria were due to an intensification of the ban on illegal logging and deforestation activities. While some reported visiting the forest to collect wild fruits and non-timber forest products, others mentioned that the ban had forced them to switch from logging and hunting to farming the agricultural land, avoiding overnight stays in the forests, patrolled by forest rangers.

Although many respondents complained there were a lot of mosquitoes around their houses, they were generally not concerned about malaria. Most respondents reported sleeping under mosquito nets and using repellent to prevent mosquito bites, although some believed they got sick with malaria by drinking water from, or staying on, certain lands.

### Fever, gastrointestinal illnesses, and other health issues

Febrile illnesses, such as dengue and flu, were often cited as health issues for children, whereas adults more commonly reported stomach pain, diarrhoea, vomiting, and indigestion. Many described experiencing illnesses from insect or other animal bites, including snake bites. Concerns regarding hygiene and sanitation also emerged, as well as heavy alcohol and energy drink consumption, indicated by VMWs as the most common cause of diarrhoea, vomiting, and stomach-related illnesses in adult males. Female respondents described that they were informed about their contraceptive choices and many reported having had injections, intrauterine devices, and/or birth control pills from health centres and local NGOs.

When prompted, community members did not perceive themselves at risk of COVID-19 and most had received vaccination. One technical officer at an international organisation summarised the health concerns in remote rural communities as follows:

*“The most pressing health needs in remote communities in Cambodia… I would say… they are water-borne diseases, skin rashes, digestive problems… they are not debilitating but still a problem also because people tend not to go to the health centre for these problems. Antenatal care is also an issue… if something goes wrong, [pregnant women] cannot spend the whole day to reach the health centre. Other problems are snake bites, which result in limb losses and deaths, and also occupational accidents in farming activities and other work”* (Technical officer at international organisation, Phnom Penh, IDI)

### Health services for the communities

In Kravanh, residents could access a variety of health services, depending on their location, socio-economic status and preferences. Respondents of Por ethnicity mentioned that traditional healers were still popular in their villages but were less common than in the past. In all villages, VHSGs, VMWs and other volunteers supported by international organisations offered general health education or specialised services for malaria, tuberculosis, maternal health, and nutrition, linking local communities with the public health sector.

The local public health centres and the district hospital provided basic medical services, including maternal care, vaccinations, and treatment for common illnesses. Poor community members explained they were eligible for various forms of subsidised assistance, including free health care. Pregnant women, mothers and children under two years of age were entitled, under a social assistance scheme for the population with low socioeconomic status, to cash transfers to attend counselling visits and check‐ups at the health centres and hospitals:

*“When my daughter gave birth at the district hospital, the hospital didn’t charge anything because we have a poor identification card (Poor 1 card) and we get 200,000 Riels (50 USD) and also 5,000 Riel (1.25USD) a day.”* (Village 2, female community member, FGD)

Despite these opportunities, distance, travel times, and associated costs were often described as significant barriers to accessing care at public health facilities:

*“The majority of the community members are poor. For example, if someone falls ill at night, our only means of transportation is motorcycle. If there is no motorcycle for some families, this is a big challenge. Our community is far from a health facility and the road condition is not good.”* (Village 1, male community member, FGD)

Community pharmacies and private clinics were often a preferred care option, delivering more personalized services and sometimes better-equipped facilities, though they were described as costly and not accessible to all:

*“It is difficult sometimes to get treatment from the health centre after showing the poor identification card (a social assistance scheme) since there are not enough staff for consultation, drugs and equipment at the facility. So I went to a private clinic and I paid a lot”* (Village 1, female community member, IDI)

### The VMW programme: Views, experiences and challenges

All interviewed VMWs engaged in agriculture as the main source of income. Many of them were appointed as VMWs because they were involved in other roles in the communities (such as a village chief, deputy chief or a member of the village committee) while others were selected by village authorities:

*“The village chief told me many times about this VMW position: ‘if you can join, you can help the community and control the spread of the disease’… I talked to my family and accepted the position.”* (Village 4, female VMW, IDI)

Following recruitment, VMWs received malaria training from various providers, including the Kravanh health centre, the provincial and district hospitals, and international organisations. Their work schedule was flexible, mainly involving door-to-door visits in their catchment area, management of suspected malaria cases, and attendance at monthly meetings in the health centre. MMWs were tasked with active case detection in forest areas and additional activities such as chemoprevention among forest workers. In addition to the malaria programme, some were involved in other community health programmes supported by the World Health Organization (WHO), Food and Agriculture Organization (FAO), and other international organisations. Most VMWs were also active in the VHSGs, assisting health centres in vaccination and health information programmes. In these multiple roles, they were seen as a focal point for immediate care in the communities.

The interviewed VMWs were generally happy about their work with the malaria programme, willing to undertake new roles, and took pride in helping their communities while some complained that social status and hierarchies could undermine acceptance by community members, especially when VMWs were perceived to be poorer than their clients. The contribution of VMWs to malaria control was recognised in the FGDs and individual interviews although financing was often cited as a significant challenge to programme implementation. For VMWs, sources of income included their monthly allowance (which was reported to be around 20 USD), irregular incentives for participation in health or rural development activities, and informal business activities, such as the sale of medicines. For example, one VMW who was also involved in a reproductive health programme explained how she would make profit selling contraceptive pills to villagers. Yet most VMWs complained about scarce remuneration and some reported that incentives had reduced recently due to the winding down of malaria projects. This was often cited as a major challenge to programme implementation, resulting in limited availability of VMWs, often busy with other income-generating activities:

*“I tried to seek help from the VMW, but he was always busy… he is a farmer and has other businesses too…The VMW comes to our house often, sometimes once every four or five days… When he comes to our house, he always explains and provides health information. And he says that if anyone in the household has any diseases or our child is old enough for vaccination, we should see a doctor as soon as possible…. But he is busy with other jobs, so he cannot visit us often…his supervisors should provide him with a daily wage and refund travel costs. Sometimes they* [VMWs] *do not have the money to buy food, do not eat enough and have no motorbikes. This affects the quality of health services provided by VMWs”* (Village 1, female community member, IDI)

### Prospects for the development of community care

There was a consensus among domestic and international stakeholders that VMWs remain a critical health system asset to support malaria control and elimination. Programme managers and international stakeholders stressed that VMWs should continue malaria services, including active surveillance and case management in low-endemic areas targeted for elimination:

*“VMWs are still really important for elimination to conduct surveillance and monitor the situation to prevent the parasite reintroduction… and VMWs living in remote areas are really important to find new cases”* (Respondent from an international organisation, Phnom Penh, IDI)

Concurrently, there was wide agreement in the communities that VMWs should provide a more comprehensive package of services, including treatment for common illnesses, monitoring of blood pressure and blood sugar levels and relief of general symptoms, such as headache, fever, and stomach pain through medication.

*“If there is no more malaria disease, the VMWs should provide more services like for non-communicable disease, medicine for fever, flu, and other diseases that need basic treatment, so we don’t need to go to the district hospital or clinic. We can be relieved and cured. There are no health posts or health centres in our area… some families don’t have motorcycles, it’s hard for them to reach health facilities on time”* (Village 5, male community member, FGD)

*“We really need extra health services in our community as soon as possible*. *We are happy with the services that the workers have provided so far*. *They are fast learners and they should get training for other services in the future”* (Village 3, male community member, FGD)

Similarly, programme managers and international stakeholders often stressed that VMW services should be expanded to ensure continued uptake and relevance. Suggestions for add-on activities included adoption of new tests for dengue and chikungunya, and the prevention and monitoring of non-communicable diseases, with careful consideration of eligibility and training, alignment with national health policy on primary care and non-communicable diseases (NCDs), and recognition that expansion of VMWs should not divert resources away from local health centres.

*“I think dengue and chikungunya can definitely be [within] the remit of VMWs like any diagnosis that doesn’t require a medical or nursing background… a diagnosis that is based on either a binary yes/no diagnostic test or you need to refer to the health centre. This is definitely something that the VMWs could do well… a lot of them are very quick in picking up technologies although we have a range of ages and a range of abilities so we should reassess the suitability of VMWs depending on the technology. Some of the older VMWs may not be suitable”* (Respondent from an international organisation, Phnom Penh, IDI)

*“We could do a pilot in one province*. *We will start redesigning the health centre to include chronic care services*. *This implies building the workforce and strengthening the public health functions*. *And Village Malaria Workers–which one day will be called Village Health Workers–will also support NCD intervention and chronic care*, *at the community level”* (Respondent from an international organisation, Phnom Penh, IDI)

*“I would want to make sure that we don’t need to rely too heavily on community health workers as a system to improve health in Cambodia… I wouldn’t want to substitute improving and investing in health facilities with reliance on VMWs… VMWs should be more of a support to this… VMWs can do many things like blood pressure monitoring*. *But I wouldn’t want them to substitute what trained health care professionals can do”* (Respondent from an international organisation, Phnom Penh, IDI)

Although there was optimism about these opportunities, concerns were raised about the availability of financial support for new roles and activities that extend beyond the remit of existing donors and funding schemes.

*“Another important part is that Global Fund funding at this level won’t last forever so the VMWs need to be integrated in the wider health system at some point and financed more by their own ministries of health”* (Consultant, Bangkok, IDI)

*“I think it is also dependent on what the donors propose like if Global Fund says we really think we would like to keep the village malaria worker network transformed into a community health worker network*, *that would be a strong incentive for governments to go along with that”* (Researcher, Bangkok, IDI)

When discussing prospects for domestic funding, provincial health managers considered scaling down certain programme components, such as shifting from monthly to quarterly meetings with VMWs in the health centres, while leveraging local resources for additional support. A senior health manager mentioned ongoing discussions between the Ministry of Health and the Ministry of Interior regarding the potential involvement of communes, which could allocate part of their budgets to community-based health services. Funding from communes was seen as an opportunity to advance administrative decentralization and enhance local ownership of these services. It was also suggested that VMWs could be upgraded into multi-purpose healthcare workers in the short to medium term, and eventually integrated into the existing community health structures–particularly the VHSGs–to provide a single point of care in the communities, as outlined in 2021 roadmap document issued by CNM [26].

*"To ensure the sustainability of community-based services, we plan to collaborate with the commune councils, as each commune has an annual budget. We try to highlight the benefits of investing in health services for their communities. Commune councils could allocate part of their budgets toward the health sector in their respective areas. To achieve this initiative, we must work closely with the Ministry of Interior."* (Programme manager, Phnom Penh, IDI)

*“In the mid-term review of the malaria strategy*, *we discussed low malaria transmission and how the community network can be sustainable*. *We have a roadmap to integrate village malaria workers in the communes by 2025 into the health system not only for malaria but also for other health diseases”* (Programme manager, Phnom Penh, IDI)

## Discussion

Our study explored views and experiences about malaria and other health concerns in rural communities in Cambodia, the current implementation of VMW programmes, and opportunities for, and challenges to, sustaining and expanding their roles. In summary, our findings further highlight the essential role of VMWs in the process of malaria elimination through their assistance in active disease surveillance, distribution of LLINs, health information, case management and referral. We also found that forest activities had reduced in the study locations but were still common. In addition, residents were often reluctant to visit public health facilities and tended to prioritise the private sector, where higher costs are incurred. In these settings, community-based services have the potential to bridge the gap between the communities and the public health sector by offering accessible, affordable, and culturally appropriate care.

The Cambodian National Malaria Programme has recognised the value of VMWs in the malaria elimination strategy. The national “Intensification Plan” to reduce malaria among high-risk populations, published in 2018 by CNM [27], emphasises the need for continued involvement of VMWs and recommended hiring additional MMWs to monitor forest workers and mobile populations in disease hotspots as well as an expansion of activities including preventive chemotherapy [28]. Other potential interventions that have been tested or explored include intermittent preventive treatment for forest workers (IPTf), involving prophylactic administration of antimalarial drugs at regular intervals, which was undergoing a pilot study during 2019–2021 [29]. In addition, testing for Glucose-6-Phosphate Dehydrogenase (G6PD) deficiency to safely administer the radical cure for *P*. *vivax* cases [30], which have contributed 90% of malaria burden in Cambodia since 2020, provides an opportunity for VMWs to fill in the gap in community case referral. This is crucial in remote communities, as a feasibility study of implementing new G6PD testing at health centres in 2021 suggested that only half of the eligible patients visited the health centres for G6PD testing after they were diagnosed with malaria by VMWs on the communities [30]. There is thus a clear potential to expand the role of VMWs to not only refer vivax patients to the health centres, but also in providing an understanding of G6PD testing, particularly on the benefits and sides effects from primaquine intake in order to keep the patients better informed about their health conditions and treatment options.

At the same time, our study documents a clear need for rethinking the vertical VMW model to ensure relevance in the communities and their changing healthcare needs, including treatment for common illnesses, monitoring of blood pressure and blood sugar levels and relief of general symptoms. As stakeholders discussed, VMWs could be merged with existing, locally owned community health structures, particularly the VHSGs, providing additional basic services for the management of infectious and non-infectious diseases. Most VMWs in our study, and as described elsewhere in Cambodia [31], were already members of the larger VHSG. Additionally, some were involved in other programmes. Thus, merging these roles into a single appointment could not only streamline healthcare delivery but also enhance the overall effectiveness and sustainability of community health initiatives.

Demands for a more comprehensive package of community-based services have been reported in Cambodia and elsewhere in the region [10, 22, 32]. For example, a study associated with an antimalarial prophylaxis trial in Northeast Cambodia identified community demands for the management of body aches, minor wounds, gastritis, diarrhoea, and vitamin deficiency [33]. Other vector borne diseases, such as dengue, neglected tropical diseases and soil-transmitted helminths, were identified as priority issues in another study in Cambodia [34]. In Southeast Myanmar, flu, diarrhoea, skin infections and tuberculosis were identified as priority diseases in the communities with recommendations to incorporate preventative and curative care into the current malaria volunteer model [35]. Evaluations of expanded programmes have also been conducted, with mixed experiences of subsequent attendance rates by febrile patients [36, 37]. Strategies to redesign the VMW model beyond malaria could learn from these approaches to adapt successful packages of roles to local demand, and to determine the effectiveness of the integrated services over time.

Despite these opportunities, dependence on external aid was often mentioned as a significant challenge to programme sustainability and further development, as documented in other studies of community health workers worldwide [38]. The vertical malaria programme heavily relies on external financial and technical support. Thus, wider political advocacy beyond CNM and ongoing support from implementing local departments and NGOs is necessary to secure adequate resources. As responsibilities shift closer to the community level due to the ongoing process of decentralisation, it is crucial to ensure that local structures are allocated the necessary resources and authority to manage these programmes effectively. This includes securing funding, building local capacity, and fostering collaboration among various stakeholders. Such efforts are vital to transition from a model dependent on external inputs to one that is integrated into the decentralised health system, thereby enhancing resilience and ensuring that malaria control remains a priority even as external support fluctuates. Taking all this into account, recommendations for VMW role expansion and integration are summarised in Box 1.

**Box 1. Recommendations for the development of the VMW programme**

1. **Strategic Allocation of VMWs**: The deployment of VMWs should be carefully tailored to reflect the evolving geography of endemic malaria and access to services in Cambodia, ensuring that their roles remain relevant and effective in addressing current and emerging health challenges.
2. **Maintain Malaria Awareness in the Communities**: Despite the decline in malaria cases, community awareness of malaria remains crucial to prevent the resurgence of the disease. VMWs should continue health education campaigns to enhance understanding of malaria’s susceptibility and severity.
3. **Conduct Local Needs Assessments**: Perform rural health assessments to identify additional roles for VMWs that respond to local needs and demands for basic care, treatment, and referral services. For instance, in Pursat, health concerns that could be addressed by VMWs include digestive illnesses, case management for arboviral diseases such as dengue and chikungunya fever, maternal and child heath, hypertension and diabetes.
4. **Align Programme Expansion with the VMW Integration Roadmap**: Ensure that the roles identified from local demands align with the VMW Integration Roadmap 2021–2025, which highlights both existing and potential roles of VMWs within the Village Health Support Group (VHSG). This integration could involve a transition from the two roles of VMWs and members of the VHSG into a unified Village Health Worker, responsible for malaria and infectious disease surveillance, health promotion, management of common illnesses, NCD prevention, maternal and child health, and referrals.
5. **Leverage Ministry of Health Policies for Support**: To secure political backing, any plans for programme development should align with the broader Health Strategic Plan 2021–2030 (HSP4) and the National Multisectoral Action Plan for the Prevention and Control on Non-communicable Diseases 2018–2027, focused on cardiovascular disease, cancer, chronic respiratory disease, diabetes, and the promotion of healthy behaviour.
6. **Explore Partnerships and Cost-Sharing Models**: The program should consider partnerships and cost-sharing models with existing health programs, the local health system, or across sectors. For example, discussions with the Ministry of Interior could explore the allocation and utilization of commune budgets for health sector activities within their respective communes.

### Strengths and limitations

This study enhances understanding of the various community-based malaria workers in Cambodia’s health system and their roles in malaria control and elimination, contributing to concurrent studies across the research projects in Cambodia [10, 39, 40] and in the region [36, 41]. We interviewed key stakeholders at different health system levels, and were able to reach VMWs with diverse experiences and involvement in health programmes in the communities. We conducted team debriefs to discuss the findings and assess their validity, and stakeholder meetings to share and triangulate the preliminary findings with selected participants. Some limitations must be noted. These include limited generalizability due to the context-specific nature of the findings, and potential sample size and selection biases, which may not capture the full diversity of experiences within the programme. The study involved interactions among Cambodian and non-Cambodian researchers with different backgrounds, disciplinary expertise, and appointments. However, we cannot rule out that subjective biases of leading authors may have influenced the interpretation of data. Additionally, reliance on self-reported data introduces risks of recall and social desirability biases, also considering that the research team had established relationships with local stakeholders due to involvement in other research projects [42]. Last but not least, the study was conducted when COVID-19 control measures were still in place, resulting in limited availability of participants and limitations to field work.

## Conclusion

There is a varied demand for healthcare at the community level, covering infectious and non-infectious diseases. We found that VMWs were accessible and well positioned to meet some of the local health needs in their communities. Programmes seeking to expand the roles of VMWs should ensure basic equipment is provided for them, as well as adequate resources and supervision. To maintain domestic political advocacy and leverage potential sources of funding, any upgrades of the VMW programme should align with national health priorities, especially primary care policy and non-communicable diseases prevention, while being responsive to changing public health needs within the endemic communities.

## Supporting information

S1 Checklist Inclusivity in global research. (DOCX)

S1 Appendix In-depth interview and focus group discussion guides. (DOCX)
